# Vaccine effectiveness against influenza hospitalisation in adults during the 2022/2023 mixed season of influenza A(H1N1)pdm09, A(H3N2) and B circulation, Europe: VEBIS SARI VE hospital network

**DOI:** 10.1111/irv.13255

**Published:** 2024-02-25

**Authors:** Angela M. C. Rose, Francisco Pozo, Iván Martínez‐Baz, Clara Mazagatos, Nathalie Bossuyt, John Paul Cauchi, Goranka Petrović, Isabela I. Loghin, Roberta Vaikutyte, Silke Buda, Ausenda Machado, Róisín Duffy, Beatrix Oroszi, Jennifer Howard, Aitziber Echeverria, Cristina Andreu, Cyril Barbezange, Aušra Džiugytė, Diana Nonković, Corneliu‐Petru Popescu, Fausta Majauskaite, Kristin Tolksdorf, Verónica Gomez, Lisa Domegan, Judit Krisztina Horváth, Jesús Castilla, Miriam García, Thomas Demuyser, Maria‐Louise Borg, Irena Tabain, Mihaela Lazar, Ieva Kubiliute, Ralf Dürrwald, Raquel Guiomar, Joan O'Donnell, Katalin Kristóf, Nathalie Nicolay, Sabrina Bacci, Esther Kissling, VEBIS SARI VE network team, Itziar Casado, Camino Trobajo‐Sanmartín, Manuel García Cenoz, Nerea Egüés, Guillermo Ezpeleta, Ana Navascués, Miguel Fernández‐Huerta, Ana Miqueleiz, Carmen Ezpeleta, SiVIRA Group for Surveillance and vaccine effectiveness in Spain, Belgium SARI Surveillance Network (BelsariNet), Lucie Seyler, Arne Witdouck, Caroline Wylock, Els Van Nedervelde, Svea Geeroms, Virgini Van Buggenhout, Nathalie Bossuyt, Sarah Denayer, Cyril Barbezange, Benédicte Lissoir, Xavier Holemans, Marc Hainaut, Nicolas Dauby, Benedicte Delaere, Marc Bourgeois, Evelyn Petit, Marijke Reynders, Door Jouck, Koen Magerman, Marieke Bleyen, Melissa Vermeulen, François Dufrasne, Tanya Melillo, Stephen Abela, Gerd Xuereb, Maja Ilić, Ivan Mlinarić, Iva Pem Novosel, Petra Tomaš Petrić, Ivana Bočina, Svjetlana Karabuva, Mihaela Čikeš Šimunković, Suzana Mladinov, Mateo Ćurin, Željka Čuljak Jurić, Joško Markić, Ivana Jukić, Ina Tomas, Marija Tonkić, Odette Popovici, Catalina Pascu, Alina Ivanciuc, Iulia Bistriceanu, Sorin Dinu, Mihaela Oprea, Maria Elena Mihai, Alexandru Marin, Gratiela Tardei, Alma‐Gabriela Tudor, Emonoil Ceausu, Simin Aysel Florescu, Elena Duca, Catalina Mihaela Luca, Carmen Mihaela Dorobat, Aukse Mickiene, Monika Kuliese, Ligita Jancoriene, Birute Zablockiene, Luise Goerlitz, Ute Preuss, Barbara Biere, Dschin‐Je Oh, Janine Reiche, Marianne Wedde, Irina Kislaya, Ana Paula Rodrigues, Camila Henriques, Aryse Mello, Gergő Túri, Katalin Krisztalovics, Annamária Ferenczi, Krisztina Mucsányiné Juhász

**Affiliations:** ^1^ Epiconcept Paris France; ^2^ National Centre for Microbiology Institute of Health Carlos III Madrid Spain; ^3^ Consortium for Biomedical Research in Epidemiology and Public Health (CIBERESP) Madrid Spain; ^4^ Instituto de Salud Pública de Navarra‐IdiSNA Pamplona Spain; ^5^ National Centre for Epidemiology Institute of Health Carlos III Madrid Spain; ^6^ Sciensano Brussels Belgium; ^7^ Department for Health Regulation Health Promotion and Disease Prevention Msida Malta; ^8^ Croatian Institute of Public Health Zagreb Croatia; ^9^ St Parascheva Clinical Hospital of Infectious Diseases Iasi Romania; ^10^ Lithuanian University of Health Sciences Kaunas Lithuania; ^11^ Robert Koch Institute Berlin Germany; ^12^ National Institute of Health Dr Ricardo Jorge Lisbon Portugal; ^13^ Health Service Executive–Health Protection Surveillance Centre Dublin Ireland; ^14^ Semmelweis University Budapest Hungary; ^15^ Subdirección de Epidemiología, Dirección General de Salud Pública Servicio Extremeño de Salud Mérida Spain; ^16^ Teaching Public Health Institute of Split‐Dalmatia County Split Croatia; ^17^ Dr Victor Babes Clinical Hospital of Infectious and Tropical Diseases Bucharest Romania; ^18^ Institute of Clinical Medicine, Medical Faculty Vilnius University Vilnius Lithuania; ^19^ Dirección General de Salud Pública, Departamento de Sanidad Gobierno de Aragón Zaragoza Spain; ^20^ Department of Microbiology and Infection control UZ Brussel Brussels Belgium; ^21^ “Cantacuzino” National Military–Medical Institute for Research and Development Bucharest Romania; ^22^ European Centre for Disease Prevention and Control Stockholm Sweden

**Keywords:** hospital, influenza, SARI patients, test‐negative design, vaccine effectiveness

## Abstract

We conducted a multicentre hospital‐based test‐negative case–control study to measure vaccine effectiveness (VE) against PCR‐confirmed influenza in adult patients with severe acute respiratory infection (SARI) during the 2022/2023 influenza season in Europe. Among 5547 SARI patients ≥18 years, 2963 (53%) were vaccinated against influenza. Overall VE against influenza A(H1N1)pdm09 was 11% (95% CI: −23–36); 20% (95% CI: −4–39) against A(H3N2) and 56% (95% CI: 22–75) against B. During the 2022/2023 season, while VE against hospitalisation with influenza B was >55%, it was ≤20% for influenza A subtypes. While influenza vaccination should be a priority for future seasons, improved vaccines against influenza are needed.

## 1 BACKGROUND

In February 2022, the World Health Organization (WHO) recommendations for the northern hemisphere 2022–2023 influenza season for trivalent influenza vaccines were to include an A/Wisconsin/588/2019 (H1N1)pdm09‐like virus, an A/Darwin/6/2021 (H3N2)‐like virus and a B/Austria/1359417/2021 (B/Victoria lineage)‐like virus. Quadrivalent vaccine was recommended to contain the above three viruses plus a B/Phuket/3073/2013 (B/Yamagata lineage)‐like virus.^1^ Egg‐based vaccines should contain the same last two viruses, in addition to an A/Victoria/2570/2019 (H1N1)pdm09‐like and an A/Darwin/9/2021 (H3N2)‐like virus.^1^ Within the European Union/European Economic Area, an early seasonal epidemic threshold (10% positive sentinel specimens) was crossed by week 45, peaking at week 51, 2022.^2^ There was co‐circulation of influenza A(H3N2), A(H1N1)pdm09 and B viruses, initially predominated by A subtypes, although patterns of dominance varied across countries.^3^ There were hospitalised severe acute respiratory infection (SARI) cases (occurring mainly in older adults) caused by infection with each of these influenza types; however, ultimately, over the 2022/2023 season, influenza A(H1N1)pdm09 predominated among SARI patients.^2^

**FIGURE 1:**
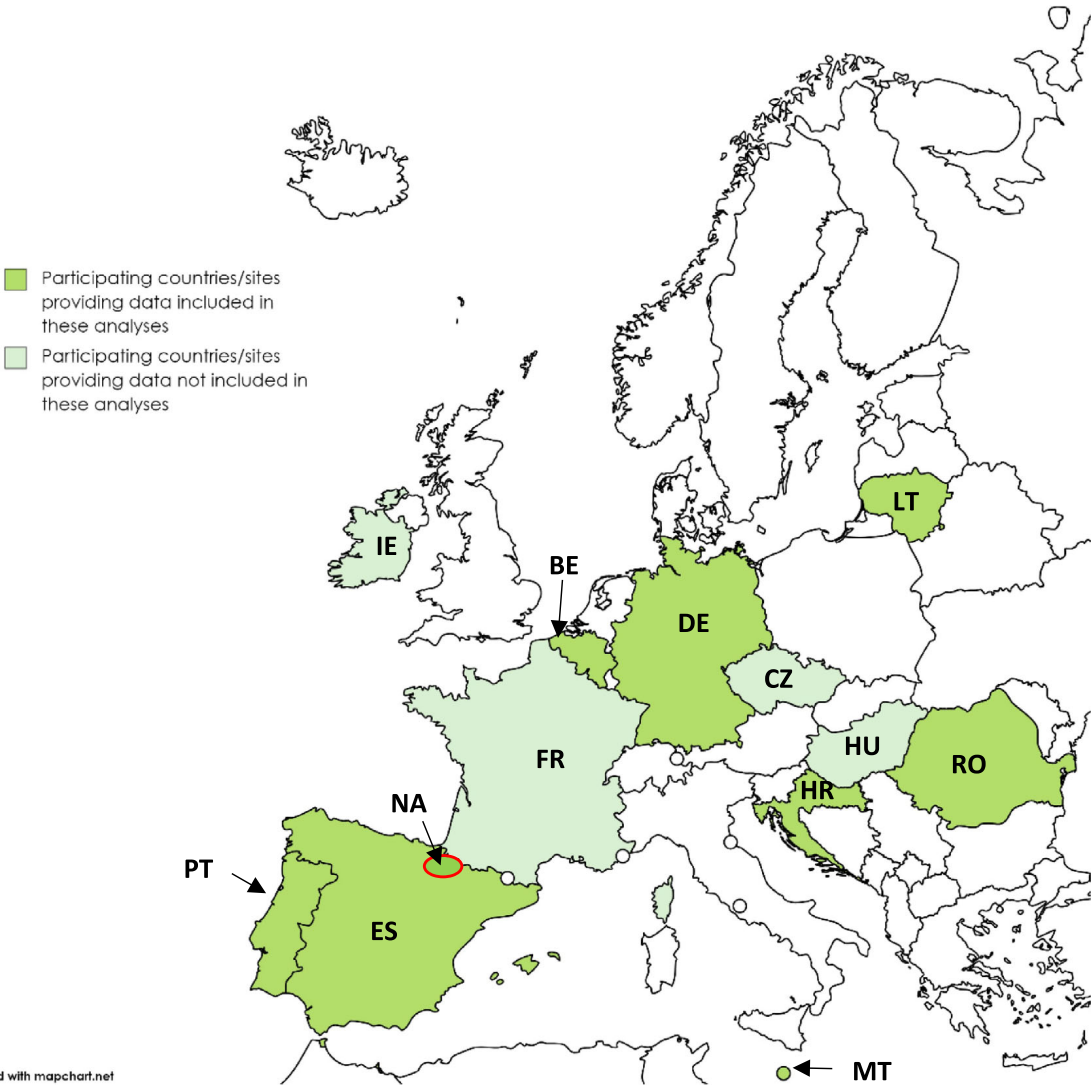
Countries and study sites^1^ participating in the Vaccine Effectiveness, Burden and Impact Studies (VEBIS) hospital network by provision of data for this analysis, Europe, 2022–2023 influenza season.^1^ Participating sites: Belgium (BE), Croatia (HR), Czechia (CZ), France (FR), Germany (DE), Hungary (HU), Ireland (IE), Lithuania (LT), Malta (MT), Portugal (PT), Romania (RO), Spain (11 regions: ES; Navarra region: NA). Included in this analysis: BE, ES, DE, HR, LT, MT, NA, PT, RO.

We pooled data from nine study sites in eight European countries participating in the ECDC Vaccine Effectiveness, Burden and Impact Studies (VEBIS) SARI vaccine effectiveness (VE) hospital network^3^ (Figure 1) with sufficient data to estimate influenza VE against circulating strains. We estimated VE against hospitalisation due to PCR‐confirmed influenza among SARI patients swabbed between 1 October 2022 and 15 May 2023.

## 2 STUDY DESIGN AND DESCRIPTIVE RESULTS

Participating sites in the network use a common generic protocol^4^, ^5^ and the test‐negative case–control design.^6^ Hospital teams collect demographic, clinical and influenza vaccination information via questionnaire, electronic medical records, vaccine registries or patient interview. We defined a SARI patient as an individual hospitalised for ≥24 h with at least one systemic symptom or sign: fever or feverishness, malaise, headache or myalgia or deterioration of general condition (asthenia, loss of weight or anorexia, confusion or dizziness) and at least one respiratory symptom or sign (cough, sore throat or shortness of breath) at admission or within 48 h after admission. Patients were excluded if their symptoms started (or clearly worsened, if chronic) more than 7 days before swabbing.

Cases were SARI patients PCR‐positive and controls PCR‐negative for influenza within 48 h of admission. We estimated VE overall, by age group (0–17, 18–64, ≥65 years), by target group for influenza vaccination according to each country's specific recommendations and by presence of at least one versus no chronic condition.

We included 5547 SARI patients aged ≥18 years (886 cases of any influenza, 4661 test‐negative controls) from 38 European hospitals, in nine of 13 participating study sites (Figure 1) providing sufficient data for this period.

Sixty‐five per cent of cases and 76% controls were aged ≥65 years, with controls having a median age of 72 years [inter‐quartile range (IQR): 56–81 years]; cases, 76 years (IQR: 65–85). Fifty‐one per cent of cases and 49% controls were female; 68% of cases and 72% of controls had at least one of the five commonly collected chronic conditions (diabetes, heart disease, lung disease, asthma, immunosuppression). Forty per cent of cases and 56% controls were vaccinated against influenza (Table 1).

**TABLE 1:** Patient characteristics of cases and controls, Vaccine Effectiveness, Burden and Impact Studies (VEBIS) hospital network, 2022–2023 influenza season, Europe (*N* = 5547).

Patient characteristic	Cases (*n* = 886)		Controls (*n* = 4661)	
Median age in years (IQR)	72 (56–81)		76 (65–85)	
	No.	%	No.	%
Age groups (years)				
18–64	313	35.3	1137	24.4
≥65	573	64.7	3524	75.6
Country‐specific vaccine target group				
No	134	15.1	502	10.8
Yes	752	84.9	4159	89.2
Sex				
Male	431	48.6	2395	51.4
Female	455	51.4	2266	48.6
At least one chronic condition^a^				
No	282	31.8	1310	28.1
Yes	604	68.2	3351	71.9
Influenza vaccination				
No	535	60.4	2049	44.0
Yes	351	39.6	2612	56.0
Influenza type and subtype				
Influenza A(H3N2)	372	42.0	NA	NA
Influenza A(H1N1)pdm09	245	27.7	NA	NA
Influenza A (subtype unknown)	162	18.3	NA	NA
Influenza B	100	11.3	NA	NA
Influenza positive, type unknown	7	0.8	NA	NA
Site/country				
Navarra	295	33.3	2346	50.3
Spain	248	28.0	1137	24.4
Romania	80	9.0	120	2.6
Belgium	72	8.1	351	7.5
Lithuania	67	7.6	117	2.5
Malta	44	5.0	288	6.2
Croatia	42	4.7	170	3.6
Portugal	22	2.5	40	0.9
Germany	16	1.8	92	2.0
Vaccine product				
Influvac tetra^b^	183	52.1	1638	62.7
Vaxigrip tetra^b^	24	6.8	166	6.4
Efluelda^b^ ^,^ ^c^ (QIV^b^)	1	0.3	5	0.2
Unknown product	143	40.7	803	30.7

Abbreviation: QIV, quadrivalent influenza vaccine.

^a^ At least one of five commonly collected chronic conditions: asthma, diabetes, heart disease, lung disease, immunosuppression.

^b^ Egg‐based, inactivated, split virion, tetra‐valent vaccine.

^c^ High‐dose vaccine.

## 3 VACCINATION DEFINITIONS AND VACCINE EFFECTIVENESS

We defined the start of the 2022/2023 influenza season in each participating site as the week number in which the first PCR‐confirmed case was reported to the study. We defined vaccination as receipt of influenza vaccine ≥14 days after the start of the current season's vaccination campaign in each participating country. Only vaccines received ≥14 days before onset were considered valid (those vaccinated 1–13 days before onset were excluded; those vaccinated on or after onset were considered unvaccinated).

**FIGURE 2:**
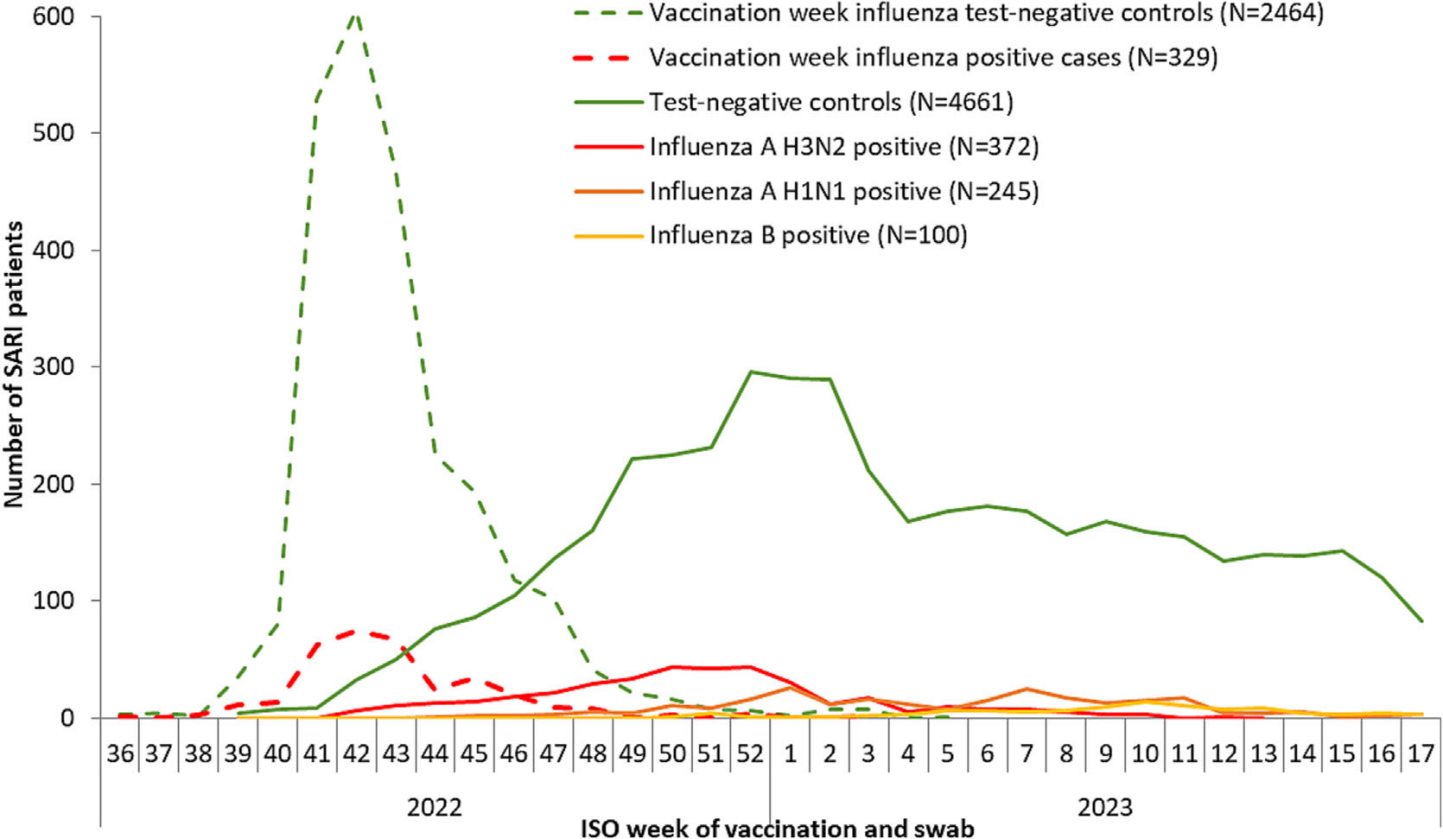
Number of severe acute respiratory infection (SARI) patients by case status and week of vaccination or swab, Vaccine Effectiveness, Burden and Impact Studies (VEBIS) in the hospital setting, influenza 2022/2023 season, Europe (*N* = 5547).

SARI patients received their influenza vaccination between weeks 36, 2022 and week 3, 2023 (cases) or week 5, 2023 (controls); Figure 2. Vaccination dates coincided with the early start of this 2022/2023 influenza season (Figure 2). The median time from vaccination to onset was 74 days for cases, 88 days for controls. Where influenza vaccine type was known (*n* = 2017/2963; 68%), all SARI patients were vaccinated with quadrivalent vaccine (Table 1).

We compared the odds of vaccination between cases and controls using logistic regression, adjusting the odds ratio (OR) of vaccination among cases and controls by age, sex and presence of at least one of the five chronic conditions listed above. We calculated VE as 1 minus the adjusted OR (expressed as a percentage). We included study site (as a fixed effect) and date of onset (as a restricted cubic spline or categorical variable using onset month) in all analyses. We excluded sites with <10 cases or controls for each influenza (sub)type‐specific analysis. We estimated interaction terms between vaccination and (1) age group and (2) chronic condition status.

Where the number of cases or controls per parameter was <10, we conducted sensitivity analysis using Firth's method of penalised logistic regression (PLR) to assess small sample bias.^7^, ^8^ We considered estimates having an absolute difference >10% between the PLR and original VE estimate indicative of small sample bias and do not show these estimates. Similarly, we do not show estimates for analyses with <20 vaccinated cases or controls. As we expected most SARI patients to have been systematically tested for both influenza and SARS‐CoV‐2, we performed sensitivity analysis excluding all controls infected with SARS‐CoV‐2.

The VE against hospitalisation with any influenza (all adults) was 27% (95% CI: 13–39), 11% (95% CI: −23–36) against influenza A(H1N1)pdm09 and 20% (95% CI: −4–39) against influenza A(H3N2). Against influenza B, VE was 56% (95% CI: 22–75) overall. Against both influenza A subtypes in patients aged ≥65 years, VE point estimates were 25%. Among those in the influenza vaccine target group, VE point estimates were 20%–22% against influenza A subtypes and 66% against influenza B. Against influenza A(H3N2), VE point estimates were 15% among those with at least one, and 32% among those without any chronic condition (*p*‐value for interaction: 0.08). Vaccinated case numbers were too small for valid estimates by age group or chronic condition status against influenza B (Table 2).

**TABLE 2:** Vaccine effectiveness against influenza hospitalisation, Vaccine Effectiveness, Burden and Impact Studies (VEBIS) in the hospital setting, influenza 2022/2023 season, Europe (*N* = 5547).

Main analysis: influenza type and subtype VE, by age group, target group and by chronic condition			
Influenza type/subtype	Vaccinated/unvaccinated cases; vaccinated/unvaccinated controls	VE^a^ (95% CI)	
**Influenza (all)**	**Nine sites** ^**b**^ **; *N* = 5547**		
All ages	351/535; 2612/2049	27	(13–39)
Age group			
18–64	49/264; 297/840	24	(−11–47)
≥65	302/271; 2315/1209	32	(16–44)
Target group for vaccination	339/413; 2525/1634	29	(14–41)
Chronic condition^c^			
No	61/221; 585/725	44	(20–61)
Yes	290/314; 2027/1324	20	(2–35)
**Influenza A**	**Nine sites** ^**b**^ **; *N* = 5095**		
All ages	328/451; 2460/1856	20	(4–34)
Age group			
18–64	44/201; 280/777	10	(−34–40)
≥65	284/250; 2180/1079	29	(12–42)
Target group for vaccination	317/358; 2378/1472	25	(9–39)
Chronic condition^c^			
No	54/180; 537/648	39	(10–59)
Yes	274/271; 1923/1208	13	(−7–30)
**Influenza A(H1N1)pdm09**	**Seven sites** ^**d**^ **; *N* = 4270**		
All ages	94/149; 2374/1653	11	(−23–36)
Age group			
18–64	17/86; 265/710	NA^e^	NA
≥65	77/63; 2109/943	25	(−12–50)
Target group for vaccination	91/109; 2294/1289	20	(−15–45)
Chronic condition^c^			
No	13/58; 522/598	NA^e^	NA
Yes	81/91; 1852/1055	8	(−33–36)
**Influenza A(H3N2)**	**Five sites** ^**f**^ **; *N* = 3471**		
All ages	161/164; 1841/1305	20	(−4–39)
Age group			
18–64	19/52; 217/540	NA^e^	NA
≥65	142/112; 1624/765	25	(−1–44)
Target group for vaccination	155/139; 1778/1039	22	(−2–41)
Chronic condition^c^			
No	27/62; 385/447	32	(−16–60)
Yes	134/102; 1456/858	15	(−15–38)
**Influenza B**	**Four sites** ^**g**^ **; *N* = 2940**		
All ages	22/63; 1863/992	56	(22–75)
Age group			
18–64	5/48; 200/440	NA^e^	NA
≥65	17/15; 1663/552	NA^e^	NA
Target group for vaccination	21/40; 1807/765	66	(31–83)
Chronic condition^c^			
No	7/34; 430/369	NA^e^	NA
Yes	15/29; 1433/623	NA^e^	NA

Sensitivity analysis: influenza type and subtype VE, after excluding controls with SARS‐CoV‐2 infection, overall, for those aged ≥65 years, and by country‐specific vaccine target groups			
Influenza type/subtype	Vaccinated/unvaccinated cases; vaccinated/unvaccinated controls	VE^**a**^ (95% CI)	
**Influenza (all)**	**Nine sites** ^**b**^ **; *N* = 4605**		
All ages	351/535; 2174/1545	31	(17–42)
Aged ≥65 years	302/271; 1927/838	36	(20–48)
Target group for vaccination	339/413; 2105/1195	33	(18–45)
**Influenza A**	**Nine sites;** ^**b**^ **; *N* = 4226**		
All ages	328/451; 2053/1394	26	(10–39)
Aged ≥65 years	284/250; 1822/744	33	(17–47)
Target group for vaccination	317/358; 1988/1071	30	(13–43)
**Influenza A(H1N1)pdm09**	**Six sites** ^**h**^ **; *N* = 3490**		
All ages	92/142; 1987/1269	9	(−29–36)
Aged ≥ 65 years	75/60; 1767/671	25	(−15–52)
Target group for vaccination	89/104; 1922/963	19	(−20–46)
**Influenza A(H3N2)**	**Five sites** ^**f**^ **; *N* = 2876**		
All ages	161/164; 1551/1000	29	(6–46)
Aged ≥65 years	142/112; 1370/549	32	(9–50)
Target group for vaccination	155/139; 1498/777	30	(7–48)
**Influenza B**	**Three sites** ^**i**^ **; *N* = 2502**		
All ages	20/55; 1608/819	62	(31–79)
Aged ≥65 years	17/14; 1431/433	NA^e^	NA
Target group for vaccination	20/37; 1560/616	66	(32–83)

^a^ Odds ratio adjusted (aOR) by country, time (restricted cubic spline of swab date or swab month as categorical variable, depending on model), age (restricted cubic spline or age as linear variable, depending on model), sex, presence/absence of chronic condition (immunocompromised, diabetes, heart disease, lung disease, asthma); VE = 1 − aOR.

^b^ Nine sites: Belgium, Croatia, Germany, Lithuania, Malta, Navarra, Portugal, Romania and Spain.

^c^ In analyses stratified by chronic condition, the adjustment for presence/absence of chronic condition was removed.

^d^ Seven sites: Belgium, Croatia, Lithuania, Malta, Navarra, Romania and Spain.

^e^ Fewer than 10 cases per parameter in the model.

^f^ Five sites: Belgium, Germany, Malta, Navarra and Spain.

^g^ Four sites: Belgium, Lithuania, Navarra and Spain.

^h^ Six sites: Belgium, Croatia, Lithuania, Navarra, Romania and Spain.

^i^ Three sites: Belgium, Navarra and Spain.

The difference between VE estimates from the main and the sensitivity analysis excluding controls with SARS‐CoV‐2 infection was ≤9%, depending on influenza (sub)type (Table 2).

## 4 DISCUSSION AND CONCLUSION

Our results from this multi‐country European hospital study suggest that, for the 2022/2023 influenza season, adult SARI patients had the highest all‐age VE against influenza B (56%). The VE was lower against influenza A(H3N2) (20%) and against influenza A(H1N1)pdm09 (11%). Point estimates were highest in older adults (≥65 years; 29% against influenza A) and those in the vaccine target group (66% against influenza B).

Despite the early season start, the vaccine coverage among controls (66% in the age group ≥65 years; data not shown) was similar to previous seasons.^9^ Our point estimates for VE against any influenza A and against A(H3N2) were similar (20%) to those found in our interim study (24%)^3^ and to our 2017/2018 end‐of‐season VE estimates for influenza A(H3N2) in a hospital setting (24%).^9^ Results from a test‐negative design case–control study in the United States and one in Italy for the 2022/2023 season reported higher overall VE results against influenza A and against A(H3N2) than ours, at 35% (95% CI: 27%–43%)^10^ and 38% (95% CI: −34–74),^11^ respectively. However, the VE in those aged ≥65 years in the Italian study was 24% (95% CI: −86–72), very close to the 25% (95% CI: −1–44) observed in our study. Although the US study included only SARS‐CoV‐2‐negative controls, their VE for influenza A was still higher than those in our sensitivity analysis including only SARS‐CoV‐2‐negative controls, although 95% CIs overlapped (26%; 95% CI: 10–39).^10^ In Europe, most circulating influenza A(H3N2) viruses belonged to haemagglutinin clade 2.2.^12^ In our study, from five sites sequencing viruses, 46/82 (56%) influenza A(H3N2) virus samples sequenced were clade 2b, 27/82 (33%) clade 2a.1b, 5/82 (6%) clade 2a.3 and 4/82 (5%) clade 2a.1 (data not shown).

Although influenza A(H3N2) was predominant among patients in primary care in Europe, influenza A(H1N1)pdm09 dominated among SARI patients.^2^ The European Centre for Disease Prevention and Control (ECDC) reported that most sequenced A(H1N1)pdm09 viruses early on in the 2022/2023 season had haemagglutinin genes in clade 5a.2, which were poorly recognised by human sera.^12^ Later circulating viruses contained several amino acid substitutions, and, indeed, most A(H1N1)pdm09 virus samples sequenced from five of our study sites were 5a.2a (55/73; 75%) or 5a.2a.1 (17/73; 23%) (data not shown). This could explain the surprisingly low VE against influenza A(H1N1)pdm09 among hospitalised SARI patients found in our study.

All 19 sequenced influenza B/Victoria viruses in our study were B/Austria/1359417/2021, matching the 2022/2023 season's vaccine strain for the northern hemisphere and likely explaining the 54% overall VE for SARI hospitalised patients against influenza B in our study.

Limitations include small sample size for younger adults (18–64 years), which, together with low vaccination coverage in this age group, led to no estimates by subtype (or for influenza B) being shown in this age group and lower precision for influenza B estimates. Multi‐country studies are inherently heterogenous, but use of a common protocol and study design, and collection of individual‐level data among our study sites, permits a larger sample size to provide robust results for continued monitoring of VE against influenza across Europe.

During the 2022/2023 season, while VE against hospitalisation with influenza B was >55%, it was ≤20% for influenza A subtypes. While better coverage for influenza vaccination should be a priority for future influenza seasons, efforts to develop improved vaccines against influenza are needed. As most hospitalised influenza patients are older adults (≥65 years), improvement of influenza A vaccines in particular is critical to help prevent severe illness among older patients.

## AUTHOR CONTRIBUTIONS

**Angela M C Rose:** Conceptualization; formal analysis; funding acquisition; methodology; project administration; software; supervision; validation; visualization; writing—original draft; writing—review and editing. **Francisco Pozo:** Data curation; investigation; methodology; project administration; resources; supervision; visualization; writing—review and editing. **Iván Martínez‐Baz:** Data curation; formal analysis; investigation; methodology; resources; writing—review and editing. **Clara Mazagatos:** Data curation; formal analysis; investigation; methodology; resources; writing—review and editing. **Nathalie Bossuyt:** Data curation; formal analysis; investigation; methodology; resources; writing—review and editing. **John Paul Cauchi:** Data curation; formal analysis; investigation; methodology; resources; writing—review and editing. **Goranka Petrovic:** Data curation; formal analysis; investigation; methodology; resources; writing—review and editing. **Isabela I. Loghin:** Data curation; formal analysis; investigation; methodology; resources; writing—review and editing. **Roberta Vaikutyte:** Data curation; formal analysis; investigation; methodology; resources; writing—review and editing. **Silke Buda:** Data curation; formal analysis; investigation; methodology; resources; writing—review and editing. **Ausenda Machado:** Data curation; formal analysis; investigation; methodology; resources; writing—review and editing. **Roisin Duffy:** Data curation; formal analysis; investigation; methodology; resources; writing—review and editing. **Beatrix Oroszi:** Data curation; formal analysis; investigation; methodology; resources; writing—review and editing. **Jennifer Howard:** Data curation; formal analysis; methodology; project administration; software; validation; visualization; writing—review and editing. **Aitziber Echeverria:** Data curation; formal analysis; investigation; methodology; resources; writing—review and editing. **Cristina Andreu Salete:** Data curation; formal analysis; investigation; methodology; resources; writing—review and editing. **Cyril Barbezange:** Data curation; formal analysis; investigation; methodology; resources; writing—review and editing. **Aušra Džiugytė:** Data curation; formal analysis; investigation; methodology; resources; writing—review and editing. **Diana Nonkovic:** Data curation; formal analysis; investigation; methodology; resources; writing—review and editing. **Corneliu Petru Popescu:** Data curation; formal analysis; investigation; methodology; resources; writing—review and editing. **Fausta Majauskaite:** Data curation; formal analysis; investigation; methodology; resources; writing—review and editing. **Kristin Tolksdorf:** Data curation; formal analysis; investigation; methodology; resources; writing—review and editing. **Verónica Gómez:** Data curation; formal analysis; investigation; methodology; resources; writing—review and editing. **Lisa Domegan:** Data curation; formal analysis; investigation; methodology; resources; writing—review and editing. **Judit Krisztina Horvath:** Data curation; formal analysis; investigation; methodology; resources; writing—review and editing. **Jesus Castilla:** Data curation; formal analysis; investigation; methodology; resources; writing—review and editing. **Miriam García‐Vázquez:** Data curation; formal analysis; investigation; methodology; resources; writing—review and editing. **Thomas Demuyser:** Data curation; formal analysis; investigation; methodology; resources; writing—review and editing. **Maria‐Louise Borg:** Data curation; formal analysis; investigation; methodology; resources; writing—review and editing. **Irena Tabain:** Data curation; formal analysis; investigation; methodology; resources; writing—review and editing. **Mihaela Lazar:** Data curation; formal analysis; investigation; methodology; resources; writing—review and editing. **Ieva Kubiliute:** Data curation; formal analysis; investigation; methodology; resources; writing—review and editing. **Ralf Dürrwald:** Data curation; formal analysis; investigation; methodology; resources; writing—review and editing. **Raquel Guiomar:** Data curation; formal analysis; investigation; methodology; resources; writing—review and editing. **Joan O'Donnell:** Data curation; formal analysis; investigation; methodology; resources; writing—review and editing. **Katalin Krisztalovics:** Data curation; formal analysis; investigation; methodology; resources; writing—review and editing. **Nathalie Nicolay:** Conceptualization; visualization; writing—review and editing. **Sabrina Bacci:** Conceptualization; visualization; writing—review and editing. **Esther Kissling:** Conceptualization; formal analysis; funding acquisition; methodology; project administration; software; supervision; validation; visualization; writing—original draft; writing—review and editing. **VEBIS SARI VE network team:** Data curation; investigation; methodology; resources; validation; writing—review and editing.

## CONFLICT OF INTEREST STATEMENT

No conflict of interest declared.

### PEER REVIEW

The peer review history for this article is available at https://www.webofscience.com/api/gateway/wos/peer-review/10.1111/irv.13255.

## ETHICS STATEMENT

The planning, conduct and reporting of the studies was in line with the Declaration of Helsinki. Official ethical approval was not required if studies were classified as being part of routine care/surveillance (Spain, Malta). In Belgium and Germany, VE is included in SARI surveillance. For Belgium, the study protocol was approved by the central Ethical Committee (CHU ST Pierre, Bruxelles) and each participating hospital's local ethical committees in 2011 (AK/12‐02‐11/4111), updated in 2014 (B.U.N. 143201215671). The German SARI surveillance was approved by the Charité‐Universitätsmedizin Berlin Ethical Board (Reference EA2/218/19). Other study sites obtained local ethical approval from a national review board (Croatia: approved 24 May 2021 and 26 January 2022, Ethics committee of the Croatian Institute of Public Health, Klasa:030‐02/21‐01/1, Ur.broj:381‐15‐21‐7; Klasa:030‐02/21‐01/1, Ur.broj:381‐15‐22‐14; Lithuania: approved by Lithuanian Bioethics Committee on 27th May 2020, and later permission extended for the study period, No. L‐20‐3/1; Navarra: PI2020/45; Portugal: approved 19 January 2021 by the Ethics Committee of Instituto Nacional de Saúde Dr Ricardo Jorge, no registration number given); Romania: approved by the Ethics Committee of the Ministerul Apărării Naionale Institutul Naional de Cercetare pentru Dezvoltare Medico‐Militară ‘Cantacuzino’ for the period 2022–2023, No. CE199/2022.

## Data Availability

Data subject to third party restrictions. The data that support the findings of this study are available from ECDC. Restrictions apply to the availability of these data, which were used under licence for this study. Data are available from the authors with the permission of ECDC and from all sites whose data are included.
